# Probing the seismic cycle timing with coseismic twisting of subduction margins

**DOI:** 10.1038/s41467-022-29564-2

**Published:** 2022-04-08

**Authors:** F. Corbi, J. Bedford, P. Poli, F. Funiciello, Z. Deng

**Affiliations:** 1 Istituto di Geologia Ambientale e Geoingegneria – CNR c/o Dipartimento di Scienze della Terra, Sapienza Università di Roma, Rome, Italy; 2 Helmholtz Centre Potsdam - GFZ German Research Centre for Geosciences, Potsdam, Germany; 3 Université Grenoble Alpes, CNRS, ISTerre, Grenoble, France; 4 Università “Roma TRE”, Dip. Scienze, Laboratory of Experimental Tectonics, Rome, Italy

**Keywords:** Seismology, Tectonics, Geophysics, Geodynamics

## Abstract

Assessing the timing of great megathrust earthquakes is together crucial for seismic hazard analysis and deemed impossible. Geodetic instrumentation of subduction zones has revealed unexpected deformation patterns at subduction segments adjacent to those that hosted recent mega-earthquakes: coastal sites move landward with faster velocities than before the earthquake. Here, we show observations from the largest and best-monitored megathrust earthquakes, and from a scaled analog model, to reveal that these events create coseismic and postseismic deformation patterns typical of a complete gear-like rotation about a vertical axis, hereafter called twisting. We find that such twisting alters the interseismic velocity field of adjacent subduction segments depending on the time since the last earthquake. Early interactions accelerate while late interactions decelerate local kinematics. This finding opens the possibility of using megathrust earthquakes, the characteristics of the twisting pattern, and the ensuing geodetic velocity changes, as a proxy for estimating the timing of the seismic cycle at unruptured segments along the margin.

## Abstract (web summary)

Satellite geodesy and downscaled laboratory experiments reveal that great subduction earthquakes trigger step changes in kinematics of neighboring segments. This signal is potentially informative of the timing of the seismic cycle.

## Introduction

The deformation pattern during the seismic cycle of subduction megathrusts has been illuminated by satellite geodesy and consists of relatively long interseismic phases characterized by landward geodetic motions interrupted by sudden coseismic seaward motions of stations that are installed on the surface of the upper plate. Between these two phases, subduction zones are subject to megathrust relocking and postseismic mantle relaxation which manifests as opposing motion of coastal and inland sites^1,2^. This relatively simple model is complicated by transient perturbations that introduce non-steadiness in the time series. Such perturbations act at various temporal and spatial scales: slow-slip occurring over days-weeks and tens-hundreds of km-scale^3,4^, while years-long accelerations over hundreds to thousands of kilometers indicate gradual changes in coupling, triggered changes in plate interface locking, and transient plunging of slabs^5–8^.

A recently discovered signal associated to great megathrust earthquakes is the postseismic acceleration of landward motion of geodetic sites located at adjacent (receiver) megathrust segments, i.e., regions of the subduction margin located tens to few hundreds of km away from the ruptured region along the trench parallel direction^9^. Clear examples include the 2011 Tohoku magnitude Mw 9.0 earthquake, where coastal sites in Hokkaido experienced faster landward motion with respect to the pre-earthquake kinematics^9,10^ and the 2010 Maule Mw 8.8 earthquake^2,9–11^ where the changes of pre-to-post earthquake surface velocities show a gear-like pattern, with coastal sites located at the sides of the ruptured region showing enhanced landward motion. A similar pattern, although less pronounced in amplitude, has been found for relatively smaller megathrust events occurred along Chilean (i.e., the 2014 Iquique Mw 8.2 and the Illapel earthquakes), Sumatra (2007 Bengkulu Mw 8.4 earthquake), and Japan (2003 Tokachi-oki Mw 8.3 earthquake) margins^9^. Several mechanisms have been proposed to explain the velocity increases: an increase of megathrust coupling^2^, postseismic slab acceleration^10^, in-plane bending of the overriding plate^12^, and continental-scale viscoelastic mantle relaxation^2,12^.

The opposite scenario (i.e., postseismic deceleration) is also apparent in paleogeodetic data. Coral microatols, living just below the intertidal zone, can be considered analogous to vertical component GNSS stations with annual time resolution. The cyclic alternation of landward interseismic- and seaward coseismic horizontal dislocation captured by modern geodesy is recorded by microatols from the Mentawai islands (West Sumatra) as interseismic submergence and coseismic emergence^13^. At the southern margin of the Mw 8.7 earthquake that struck the Mentawai segment of the Sunda megathrust in 1797, sites that were subsiding interseismically appear to have undergone a slowdown of factor ~2 right after that earthquake occurred, likely due to a decrease of megathrust coupling^14,15^.

The lack of a model capable of reconciling apparently opposite behaviors (i.e., accelerated or decelerated kinematics of the receivers) prompts a re-examination of segment interaction dynamics, a process deemed crucial for seismic hazard assessment, as it can influence the temporal progression of subsequent great megathrust earthquakes^16–18^. Unfortunately, the paucity of observations from natural cases and the multiple- interrelated contributions (e.g., afterslip, postseismic relaxation) that act at different spatial and temporal scales hinders discriminating between different driving mechanisms.

Here, we first look at the margin-wide displacement patterns from the best-monitored megathrust earthquake, i.e., the 2011 Tohoku-oki Mw 9.0 earthquake and then compare it alongside observations from analog subduction models to unravel the mechanisms that might control the landwards speed-up or slow-down of adjacent segments of the ruptured subduction zone. We show that mega-earthquakes create a distinct pattern at rupture edges typical of a complete rotation about a vertical axis, hereafter referred to “twisting”. We show that such twisting occurs coseismically and is sustained postseismically. Our analysis of tens of seismic cycles from the laboratory data shows that the shape of twisting contains information about the timing of the seismic cycle of adjacent subduction segments.

## Results

### Coseismic and postseismic twisting in nature

**Fig. 1:**
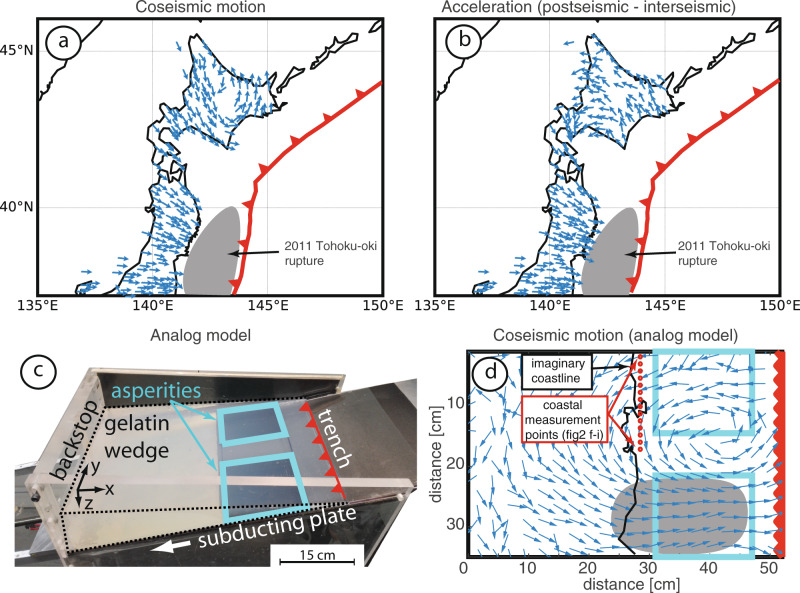
Twisting patterns in nature and in the laboratory. **a**, **b** Unit vectors show the direction of coseismic motion and acceleration from interseismic to postseismic for the 2011 Tohoku-oki earthquake recorded by permanent GNSS stations. **c** Oblique view photo of the analog model. **d** Unit vectors show the direction of coseismic motion from one analog earthquake. Cyan rectangles highlight the two asperities. Red lines indicate the trench.

Similar to previous studies^2,9,10^, we take GNSS displacement time series from before and after great megathrust earthquakes and analyze the large-scale deformation field (see the “Methods” section). The notable difference here is that we also analyze the coseismic motion in addition to the acceleration from interseismic to postseismic (i.e., postseismic acceleration). Figure 1 represents coseismic motion (panel a) and the postseismic acceleration (panel b) using velocities estimated from 2 years before and after the Mw 9.0 Tohoku-oki, Japan, 2011 mega-earthquake. A pronounced counterclockwise twisting is apparent in both cases. The vortex center is slightly closer to the hypocenter for the postseismic acceleration case. This deformation pattern appears to be asymmetric with respect to the rupture (Supplementary Fig. 1), likely because south of the Tohoku-oki rupture the coastline veers to the west and the coseismic twisting would occur offshore. Therefore, coastal sites in Shikoku and Honshu move toward the rupture coseismically and postseismically.

A similar pattern can be seen for both the coseismic and postseismic acceleration of the Mw 8.8 Maule, Chile, 2010 earthquake (Supplementary Fig. 2) where the fewer stations in the network do not allow for as precise a localization of the vortex center as in the Tohoku-oki case.

### Twisting pattern from analog subduction models

**Fig. 2:**
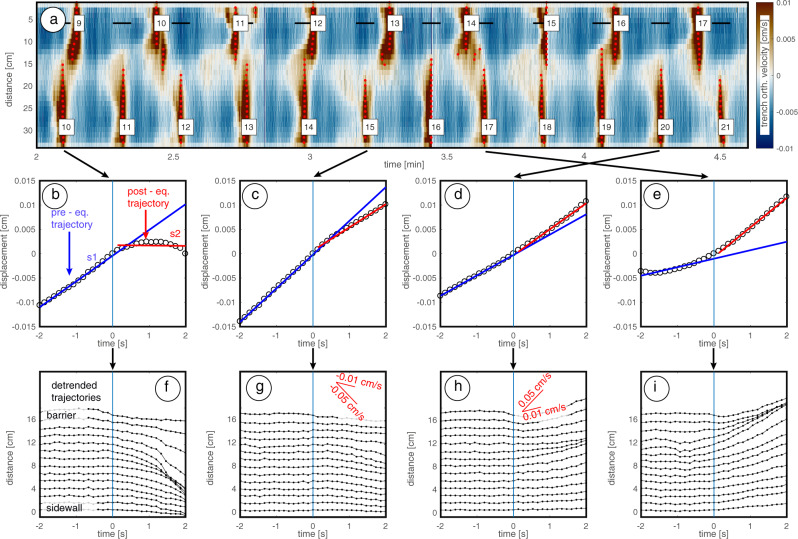
Coseismic segment interaction. **a** Time evolution of the trench-orthogonal component of the velocity field sampled along a section parallel to the trench and vertically intersecting the asperities mid-depth. Bluish and brownish colors correspond to landward and seaward velocities, respectively. Red dots represent velocity peaks, while black segments represent time windows of 4 s duration whose centers correspond to the velocity peak in the adjacent asperity. **b**–**e** Trajectories corresponding to single-pixel wide profiles at the location of the black segments shown in panel **a**. Blue and red segments represent pre- and post earthquake linear fits, respectively. Panels **b**–**e** are arranged in order of increasing Δv. **f**–**i** For the same interactions we report time series of coastal measurement points on the receiver, detrended of the pre-earthquake velocity. Red lines report reference velocity changes.

We use data from an analog seismo-tectonic model for simulating segment interaction over multiple seismic cycles under well-constrained boundary conditions^19,20^. The model consists of an elastic (i.e., storage modulus >> loss modulus^21^) gelatin wedge analog of the overriding plate underthrusted (with a velocity of 0.01 cm/s) by a rigid aluminum subducting plate featuring two rectangular velocity weakening frictional patches, hereafter referred to as asperities (Fig. 1c, d). The model creates stick-slip dynamics, with alternating phases of landward velocities (i.e., interseismic phase) and fast seaward velocities (i.e., coseismic phase; Fig. 2a). Models with two asperities host both ruptures of only one asperity (i.e., single asperity ruptures) and ruptures of both asperities (i.e., double-asperity ruptures), with distance between asperities controlling their relative proportion^19,22^. Here we selected a model configuration characterized by mainly single asperity ruptures. This condition is particularly favorable for studying the effects of mutual interaction between adjacent segments: each asperity acts alternatingly as “trigger” and “receiver”. Experimental monitoring based on the Particle Image Velocimetry (PIV) technique allows measuring the in-plane deformation with a precision of few tens of μm and resolution of few mm spacing, similarly to a spatially dense, continuous GNSS network extending also above the generally offshore seismogenic zone^23,24^ (“Methods”).

We examine the velocity changes Δv = s_2_−s_1_ (being s_1_ and s_2_ the slope of linear trajectory trend during pre- and post earthquake periods, respectively) occurring at the moment of the coseismic velocity peak in the trigger asperity (“Methods”). We observe segment interactions that lead to both a velocity reduction (i.e., Δv < 0) and increase (i.e., Δv > 0) on the receiver (Fig. 2b–e). The surface velocity fields show that: (a) the largest Δv are not recorded at the coastal sites closest to the rupture tip (Fig. 2f–i); and (b) segment interactions causing Δv > 0 and Δv < 0 on the receiver asperity have different patterns of surface displacement.

**Fig. 3:**
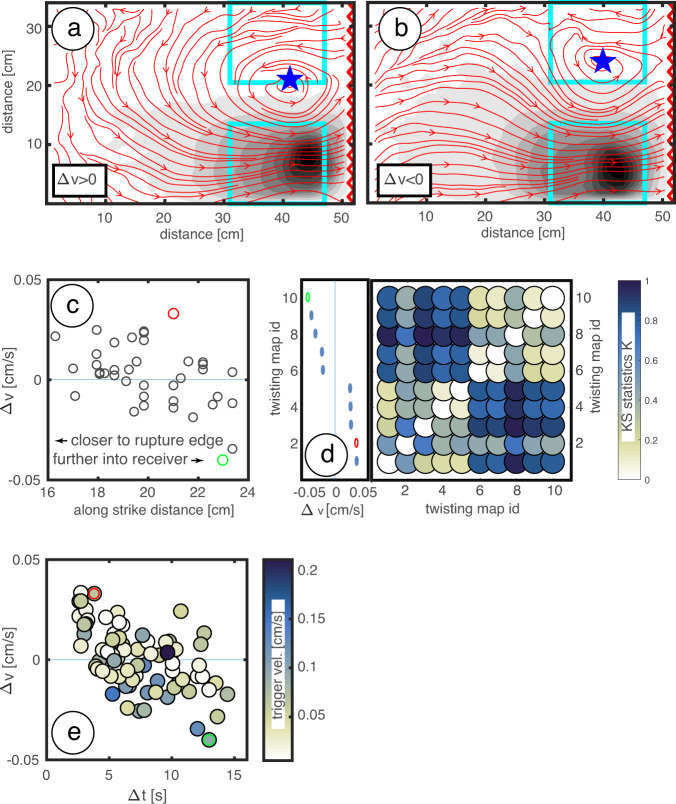
Twisting accelerates and decelerates receiver kinematics. **a**, **b** Coseismic displacement pattern highlighted by streamlines, showing twisting about a vertical axis for both positive and negative Δvs. Arrows indicate a counterclockwise circulation. The gray background shading represents the rupture (see also Supplementary Fig. 13 showing the velocity field during both interactions). **c** Bivariate plot showing the relationship between the location of twisting centers and Δv. **d** Kolmogorov–Smirnov statistic K computed for all combinations of couples of twisting maps with the largest Δv (Δv reported on the subplot to the left). K highlights the degree of similarity between couples of coseismic displacement maps. Light and dark blue color indicate similar and dissimilar couples of twisting maps, respectively. **e** Bivariate plot showing the relationship between Δv and Δt over multiple segment interactions. Circles are color-coded by the amplitude of the velocity peak of the triggering asperity. Markers highlighted by green and red circles correspond to displacement maps shown in panels **a** and **b**, respectively.

We show the pattern of surface deformation as streamlines for two interactions characterized by rupture of the southern asperity for opposite Δv polarities (Fig. 3a, b). Streamlines are constrained by the velocity field observed at the moment of the velocity peak of the triggering asperity. In both cases, the model is subject to counterclockwise twisting. However, the shape of the streamlines is different: a wide ellipse elongated in trench-orthogonal direction and rotation pole located at the edge of the receiver asperity for Δv > 0, and a smaller ellipse with the rotation pole shifted inside the receiver asperity for Δv < 0. The largest differences between deformation fields for the Δv > 0 and Δv < 0 cases are found at onland sites in the receiver region (Supplementary Fig. 3). In this area, streamlines associated with Δv > 0 are generally directed landward. Streamlines associated with Δv < 0 move toward the rupturing asperity, primarily trenchward. We verified that both the trench parallel rotation pole shift and the surface deformation pattern are not anecdotal of the two interactions shown in Fig. 3a, b, and that they are characteristic of interactions displaying the greatest positive and negative Δvs. In particular, we found an inverse proportionality between Δv and the distance of the rotation pole from the trigger asperity (Fig. 3c) and that this proportionality appears only secondly affected by differences in slip distribution of individual events (Supplementary Fig. 4). We constrain the similarity between pairs of twisting patterns using the two samples Kolmogorov–Smirnov statistic K = max(|F1(x)−F2(x)|); i.e., the maximum absolute difference between empirical cumulative distribution functions of two data vectors F1 and F2. K ~ 0 indicates similar distributions while K ~ 1 indicates dissimilar distributions. F1 and F2 in our case represent the trench-orthogonal component of displacement in the receiver region. By cross-checking all potential combinations for pairs of twisting maps, we verified that interactions with the largest Δv > 0 amplitudes are systematically similar (i.e., K ~ 0) to each other and dissimilar (i.e., K ~ 1) to interactions with largest Δv < 0, and vice-versa (Fig. 3d). Alternative methods for assessing the similarity between couples of twisting maps are reported in Supplementary Fig. 5.

## Discussion

The two end-member Δvs shown in Fig. 2b, e (corresponding to twisting maps shown in Fig. 3 panel b and a, respectively) occur very late and very early with respect to the receiver seismic cycle, while segment interactions with relatively smaller Δv occur in the middle of the cycle. A careful inspection of interseismic trajectories reveal that Δvs of similar amplitudes to those observed at the moment of asperities’ interaction manifest also at random interseismic times due to the non-stationarity of the system. Therefore, we interrogate Δv only at the moment of interaction and associate it to the corresponding twisting pattern. We consider Δv a local indicator of the receiver’s changing kinematics in response to segment interaction and the twisting pattern as a more informative proxy of the underlying interaction process. To investigate how twisting maps are representative of a given stage of the seismic cycle of the receiver segment, we compared Δv with the time interval between the beginning of the cycle in the receiver region and the moment of segment interaction Δt = t_r_−t_t_ (where t represents the experimental rupture time and the subscripts r and t refer to receiver and trigger, respectively). Here, Δt is an appropriate parameter describing timing of the seismic cycle of the receiver. We found that Δv and Δt are negatively correlated (R = 0.57) and that the amplitude of the velocity peak as well as the magnitude of triggering events play a secondary role in controlling Δv (Fig. 3e; Supplementary Fig. 6a). The latter outcome seems to contradict the direct proportionality between earthquake magnitude and amplitude of velocity changes observed in nature^9^. In the laboratory, the magnitude of triggering events determines the location along strike of vortex centers, with larger magnitudes generally associated to centers shifted farther into the receiver asperity (Supplementary Fig. 6b), but in the coastal area, where Δvs are computed, the twisting pattern is primarily controlled by the amount of shortening of the gelatin wedge.

While the twisting pattern is useful for assessing the timing of the seismic cycle of a simplified laboratory model, an increased experimental complexity is required to reasonably reproduce the real subduction environment and, in turn, to export this finding to the natural prototype. However, the identification of this general twisting pattern as an indicator of asperity maturity can be exploited in models that attempt to forecast the time remaining until failure. For example, features derived from this twisting might be useful to Machine Learning algorithms that predict the onset of fault failure in the lab and in nature^23–26^.

The time dependence of twisting helps in discriminating between different responsible driving mechanisms. Numerical models mimicking the postseismic deformation associated to large megathrust earthquakes show that a twisting pattern, similar to that one discussed in this study, may or may not appear as a consequence of viscoelastic relaxation, depending on model configuration and implemented rheologies^9,27,28^. However, a common feature of any previously modeled “postseismic driven twisting” is that it vanishes quickly in time [e.g., within 0.15–0.5 years for the Maule earthquake^28^]. On the contrary, in our analog models and in the case of the Tohoku and Maule earthquakes, adjacent segments experience a velocity step change at the moment of the triggering earthquake followed by a sustained linear motion (Supplementary Figs. 7–9). Similar sustained velocity changes have been found in earlier modeling studies and in limited natural observations^2,9,10,29^. Since there is no mantle equivalent in our analog model and gelatin rheology is predominantly elastic at the timescale of the experiment^21^, much of the observed postseismically persistent twisting must be the product of static stress changes in combination with non-linearities in the system caused by the presence of a physical frictional sliding surface, i.e., the analog megathrust. Coseismic and postseismically sustained twisting patterns, similar to those spatiotemporal features produced by our analog experiments, have been produced in numerical simulations that implement a physical frictional boundary layer as analog of the megathrust^2^, indicating that this feature is crucial for reproducing sustained kinematic changes. Since Elastic Dislocation Models EDMs neglect this frictional boundary layer, they capture the twisting pattern only partially. In particular, EDMs mimic the rotation observable at rupture edges but fail to reproduce the complete rotation characteristic of the twisting (“Methods” and Supplementary Fig. 10). Another clue testifying to the importance of the frictional boundary layer derives from Coulomb failure stress change ∆CFS. ∆CFS at adjacent segments estimated with EDMs is generally small (i.e., on the order of a few KPa^27^; comparable with atmospheric pressure fluctuations) with respect to coseismic stress drops of large subduction earthquakes (i.e., on the order of a few MPa^30^). Compared to EDMs, models that include a “physical” frictional boundary layer, such as that one implemented in ref. ^2^, predict higher ∆CFS (on the order of tens-hundreds of KPa). Although we cannot define precisely the relative importance of postseismic versus static stress change as a driver, the results of the numerical model in ref. ^2^ suggest that static stress change (increased shear stresses) is large enough to increase the degree of interplate coupling and in turn cause faster surface velocities and shortening rates^2^. Similarly in the laboratory, segment interaction might also change the gelatin-megathrust coupling (kinematic coupling ratio; sensu ref. ^31^) at adjacent segments and, in turn, tune surface velocities. Such velocity changes together with local stress loading history, result in the observed twisting pattern.

In nature assessing drivers of the observed postseismic accelerations is more complex as the subduction margin is frequently failing in different segments with different magnitude earthquakes. For instance, an adjacent segment might already be relaxing postseismically from a large event that has recently occurred in that segment, or an acceleration of an adjacent segment could be interrupted by a large event triggered nearby.

**Fig. 4:**
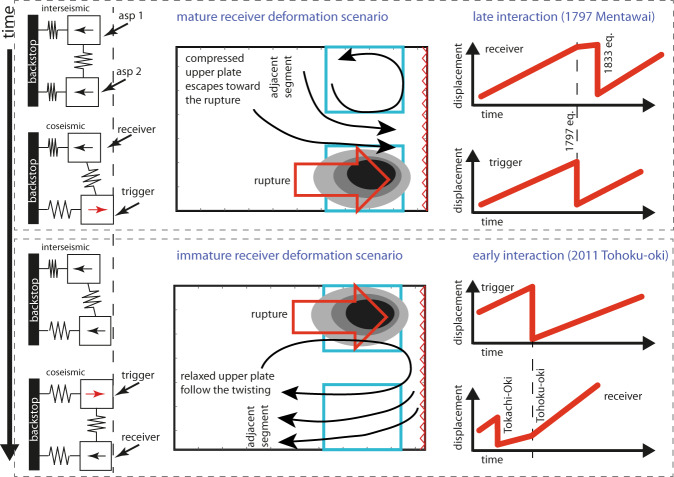
Temporal evolution of seismic cycling in mature and immature interaction scenarios. Coupled spring-slider system represents alternating trigger and receiver segments of a generic subduction zone. Failure of asperity 2 with asperity 1 close to the end of the loading phase represents a “mature” receiver. Failure of asperity 1 with asperity 2 at the beginning of the loading phase represents an “immature” receiver scenario.

Along with the analysis of velocity changes, we also provide a conceptual model to explain how the observed twisting indicates segment maturity (Fig. 4). In the double-asperity analog model, a mature (ready to fail due to being at the end of its seismic cycle), compressed upper plate confines the twisting to the offshore region. The receiver asperity is thus postseismically moving landward more slowly than expected. On the contrary, an immature (just ruptured and therefore at the beginning of its cycle), relaxed upper plate exerts no resistance against the twisting, causing the whole model deformation to coherently follow the twisting pattern and the receiver region to move landward more quickly than expected. This conceptual model implicitly proposes an upper plate compression during the interseismic period primarily controlled by persistent asperities. Therefore, the frictional properties of the plate interface, along with the compressional state of the upper plate control the appearance of the observed twisting. This conceptual model behavior finds similarities with respect to recent observations from natural subduction zones. Segment interactions caused by the Mw 9.1 Tohoku-oki 2011 and by the Mw 8.7 Mentawai 1797 earthquakes represent the two clearest end-members for accelerated and decelerated kinematics, respectively. Interestingly, the timing of the seismic cycle in those two regions matches well the inverse Δv–Δt proportionality experimentally found. The segment of megathrust facing Hokkaido island where GNSS sites experienced enhanced landward motions after the Mw 9.1 Tohoku-oki 2011 earthquake had ruptured 8 years before, during the Mw 8.3 Tokachi-oki 2003. According to paleogeodetic data, the megathrust segment facing Pangai, where reduced kinematics was caused by the Mw 8.7 Mentawai 1797 earthquake, had ruptured 94 years before in 1703. Compared to the centennial–multicentury recurrence time for large megathrust earthquakes^32^ 8 or 94 years would correspond to a relatively short- and long-time interval between the last earthquake and the segment interaction episode, respectively. According to our framing, the accelerated and decelerated kinematics observed after the Mw 9.1 Tohoku-oki 2011 and by the Mw 8.7 Mentawai 1797 earthquakes might represent the manifestation of “immature” and “mature” interactions, respectively.

Indeed, the state of upper plate compression in natural subduction zones, on the scale of fully relaxed to fully compressed, is difficult to be quantified without multi-cycle deformation records. Therefore, the observed twisting patterns should be used alongside other methods such as historical seismicity^33^, paleoseismicity^29^, and numerical modeling^34^ in analyzing segment maturity and, in turn, in probing the seismic cycle timing.

We have shown that great megathrust earthquakes and their experimental equivalents create a distinct twisting deformation pattern that develops at adjacent segments. We demonstrated that this twisting cannot be explained by elastic dislocation modeling but is related to the amount of compression in the upper plate which, in turn, is controlled by the long-term frictional properties of the plate interface. The atypical velocities of segments adjacent to the rupture do not decay, being sustained into the postseismic phase. This feature indicates a kinematic change of the plate interface behavior due to coseismic static stress transfer. The shape of twisting has the potential to mirror the seismic maturity of adjacent asperities, contributing to defining robust time-dependent seismic hazard models. Future research and community discussion will help to establish the role of decaying postseismic processes (such as viscoelastic relaxation and afterslip) as well as the roles of frictional and rheological heterogeneities (at both the megathrust and in the upper plate) in controlling this deformation pattern.

## Methods

### Extracting velocities and displacements from GNSS time series

For the Tohoku-oki analysis, we use the F3 network solutions^35^ between 1 January 2008 and 1 January 2014. Earthquake-related steps and steady-state seasonal oscillations are removed from the time series after first being fit using a customized linear regression approach that also accounts for the secular variation in the time series^36^. From the remaining time series, the interseismic and postseismic data are each fit with a 1st order polynomial. The difference between these velocities (similar to how ∆v is calculated from the laboratory time series) is plotted in Supplementary Fig. 11. Supplementary Fig. 7 shows example time series (after removal of steps and steady-state seasonal) from which the interseismic and postseismic velocities are calculated. The Tohoku-oki coseismic displacements are taken from the series of earthquake step functions that have been isolated using the regression analysis. Since the GNSS displacement series are provided in daily sampling, the earthquake appears in the time series as occurring over 2 days. The step functions in the regression take into consideration this smearing of earthquake steps due to sampling and so the coseismic displacements are taken between 10 and 12 March 2011. The reference frame of the coseismic displacements is the International Terrestrial Reference Frame 2005 ITRF05^37^. In Fig. 1, the coseismic displacement and acceleration from interseismic to postseismic are plotted with unit vectors in order to highlight the twisting pattern.

For the Maule earthquake, we repeated this analysis using Precise Point Positioning (PPP) daily solutions between 1 June 2007 and 1 June 2012. The PPP solutions were prepared using Earth Parameter and Orbit System (EPOS) software from GFZ^38^. In order to ensure consistency in the PPP solutions the precise satellite orbits as well as clock products were taken from the GFZ 2nd reprocessing^39^ obtained by the EPOS software. The EPOS software and GFZ reprocessing orbit and clock products can guarantee the best possible PPP solutions. In the PPP data processing, the basic observations are different ionosphere-free linear combinations from GPS L1 and L2 signal. The absolute phase center offset and variations were applied. The station coordinates were corrected with the FES2004 ocean tide loading model^40^. Apriori zenith hydro-static delay is obtained using the Global Pressure and Temperature model (GPT2) and Vienna mapping functions in a grid file database. The standard deviation of the daily positions per measurement is about 2 mm. In the vertical direction, the standard deviation is about 5 mm^41^. Coseismic displacements (between 26 and 28 February 2010) and the acceleration from interseismic to postseismic are shown in Supplementary Fig. 9. The reference frame of the coseismic displacements is the ITRF14^39^.

### Extracting velocities and displacements from digital images

Experimental monitoring is performed with a CCD camera (ALLIED-PIKE) which acquired a sequence of top view, high-resolution (1600 × 1200 pixels^2^, 8 bit, 256 gray levels, 7.5 frames per second) digital images of the model surface (x–y plane). Incremental surface displacement between subsequent images is then calculated with MatPIV^42^, a Particle Image Velocimetry PIV software that allows measuring deformation field with a precision of few tens of μm and resolution of few mm. Velocities of each measurement point are computed from the displacement, knowing time between consecutive frames.

### Calculation of Δv

We examine velocity changes Δv that occur above the center of the receiver asperity at the moment of the velocity peak. First, we select a 4 s long displacement time series of the target station centered at the moment of the velocity peak of the trigger asperity (Supplementary Fig. 12 shows the sensitivity to time window length). If rupture of the receiver asperity occurs within the analyzed time window the interaction is discarded. For the remaining interactions (i.e., 51 interactions over 138 analog earthquakes), we split displacement data into two halves and calculate the pre- and post earthquake velocities as slope of linear fits s_1_ and s_2_, respectively. Δv is finally defined as the difference between s_2_ and s_1_.

### Predicted surface patterns from elastic dislocation modeling

We forward model the surface predictions from the elastic dislocation model using elastic dislocation Green’s functions^43^ on a plate interface resembling a dipping subduction megathrust (Supplementary Fig. 10). The dip of the fault is 10 degrees and a coseismic slip, with radially decaying, peak magnitude of 1 m is imposed on the patches of this fault. Slip is purely dip-dip, with no strike-slip component. This forward modeling exercise serves to demonstrate that the coseismic twisting observed in natural and laboratory earthquakes cannot be reproduced by the elastic dislocation model.

## Supplementary information

Supplementary Information

## Data Availability

GNSS displacement time series (F3 solutions) can be requested from the Geospatial Information Authority of Japan (GSI). GNSS time series used for the South American analysis can be requested from Jonathan Bedford (jonathan.bedford@gfz-potsdam.de). The experimental dataset generated during the current study (i.e., digital images from the analog model) are available from the corresponding author upon request. Displacement maps dataset from the analog model, together with data description file and a Matlab script allowing to reproduce a movie of surface displacement are available open access^44^ through the GFZ Data Services.

## Code availability

GPS data were processed with the Earth Parameter and Orbit System (EPOS) software from GFZ. https://www.gfz-potsdam.de/en/section/global-geomonitoring-and-gravity-field/topics/earth-system-parameters-and-orbit-dynamics/earth-parameter-and-orbit-system-software-epos/. Customized linear regression code^36^ is available upon request from Jonathan Bedford (jonathan.bedford@gfz-potsdam.de). Digital images from the analog model were processed using MATPIV software, freely available from https://www.mn.uio.no/math/english/people/aca/jks/matpiv/.
