# Supplementary material for: Probing the seismic cycle timing with coseismic twisting of subduction margins
Source: Nat Commun. 2022 Apr 8;13:1911. doi: 10.1038/s41467-022-29564-2 (PMC8993884; doi:10.1038/s41467-022-29564-2)
Supplement: Supplementary file 1 — Supplementary Information [file 41467_2022_29564_MOESM1_ESM.pdf]

Supplementary information: Probing the seismic cycle timing with coseismic twisting of subduction margins

F. Corbi[1*], J. Bedford[2], P.Poli[3], F. Funiciello[4], Z. Deng[2]

[1] Istituto di Geologia Ambientale e Geoingegneria – CNR c/o Dipartimento di Scienze della Terra, Sapienza Università di Roma, Rome, Italy.

[2] Helmholtz Centre Potsdam - GFZ German Research Centre for Geosciences, Potsdam, Germany.

[3] Université Grenoble Alpes, CNRS, ISTerre, Grenoble, France.

[4] Università "Roma TRE", Dip. Scienze, Laboratory of Experimental Tectonics, Rome, Italy.

*Corresponding author: Fabio Corbi (fabio.corbi3@gmail.com)

Supplementary Information File: This document contains 13 supplementary figures referred by the main text and Methods of the article.

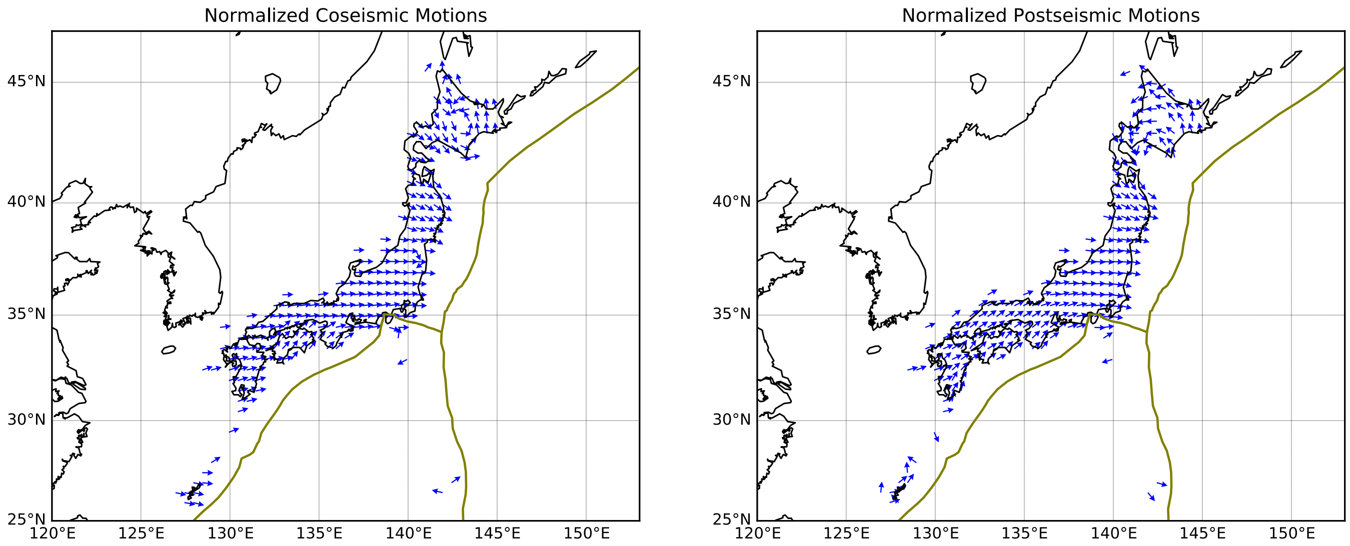

Supplementary Figure 1: Coseismic and Postseismic displacement for the 2011 Tohoku-oki earthquake. a,b Unit vectors show the direction of coseismic motion and acceleration from interseismic to postseismic for the 2011 Tohoku-oki earthquake recorded by permanent GNSS stations.

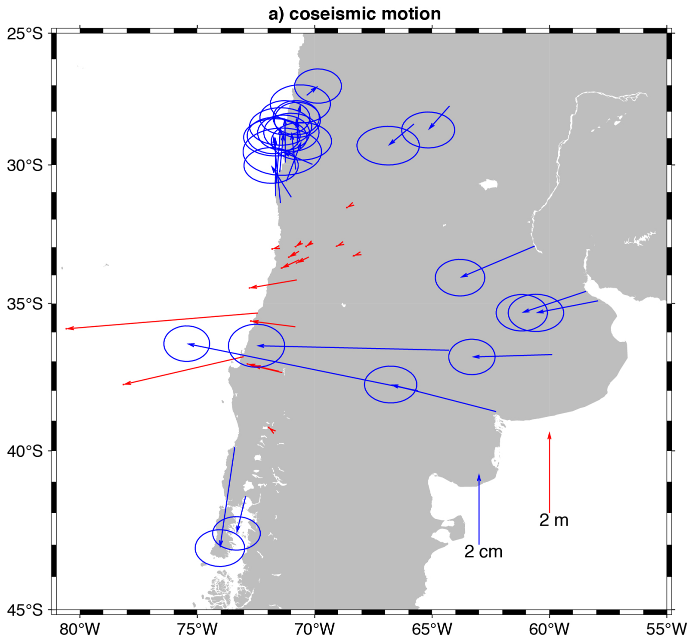

Supplementary Figure 2: Displacement of the Maule earthquake. a The coseismic motion of the Maule earthquake. **b** The acceleration from interseismic to postseismic velocity. Due to much larger displacements in the vicinity of the Maule rupture zone, we employ two colour scales. In both panels, there is a hint of the vortex-like twisting motion, but the lack of spatial coverage of GNSS stations does not show this convincingly. Dashed line indicates the trench. Ellipses represent 95% confidence.

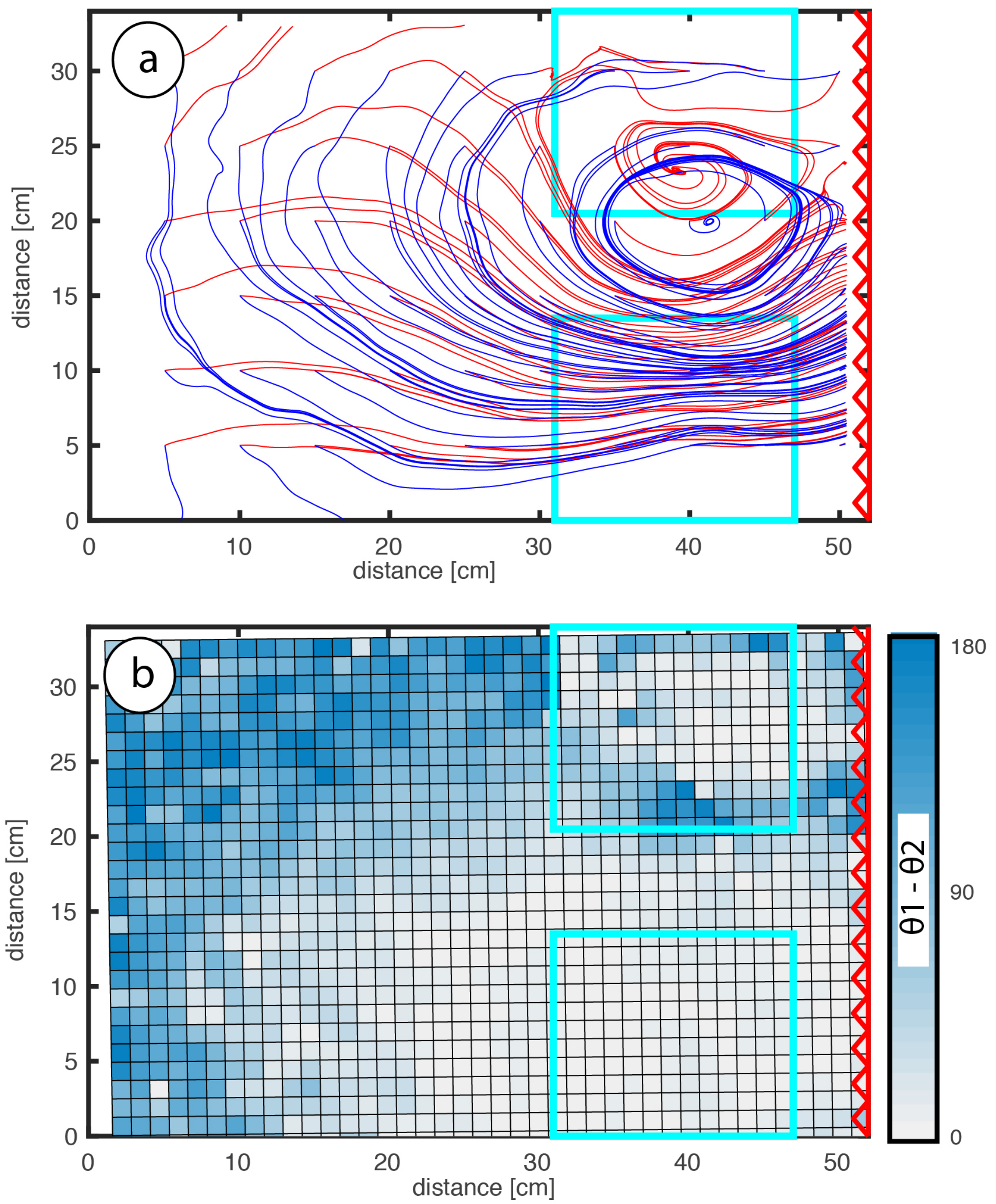

Supplementary Figure 3: Comparison between twisting maps: a accelerating (Δv>0) and decelerating (Δv>0) twisting patterns shown with blue and red streamlines, respectively. **b** map showing the difference between the strike Θ of the two maps reported in panel a. Bluish and whitish colors correspond to largest and smallest differences between the two maps, respectively. Cyan rectangles highlight the two asperities. Red lines indicate the trench. The largest differences between Δv>0 and Δv<0 twisting maps are found at onland sites in the receiver region.

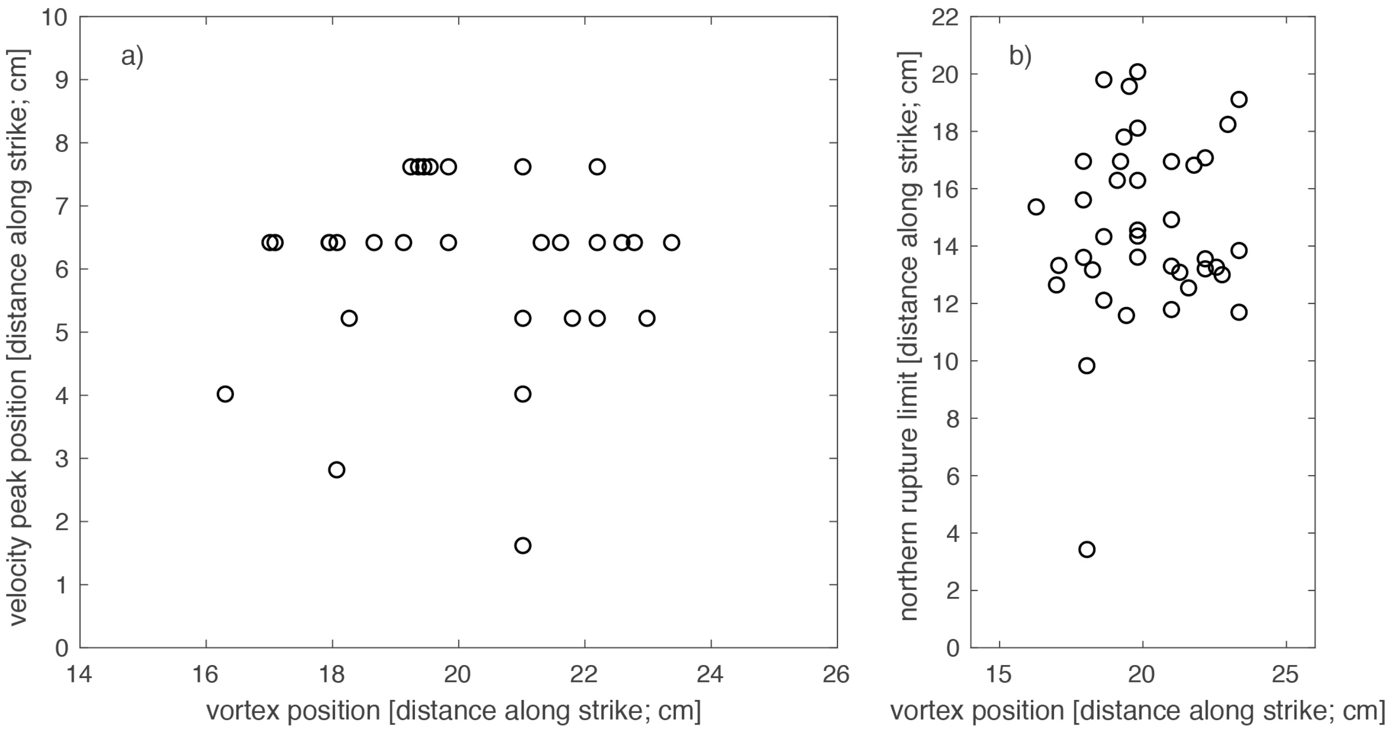

Supplementary Figure 4: How rupture characteristics influence the position of vortexes centers: a Bivariate plot showing the position of twisting centers with respect to the along trench position of the rupture velocity peak of the rupture. **b** Bivariate plot showing the position of twisting centers with respect to the along strike position of the rupture tip (limit closer to the vortex center; e.g., northern rupture limit in Figure 3a,b). The lack of correlation in panel a and b suggest minor influence of rupture characteristics on the position of the vortexes centers.

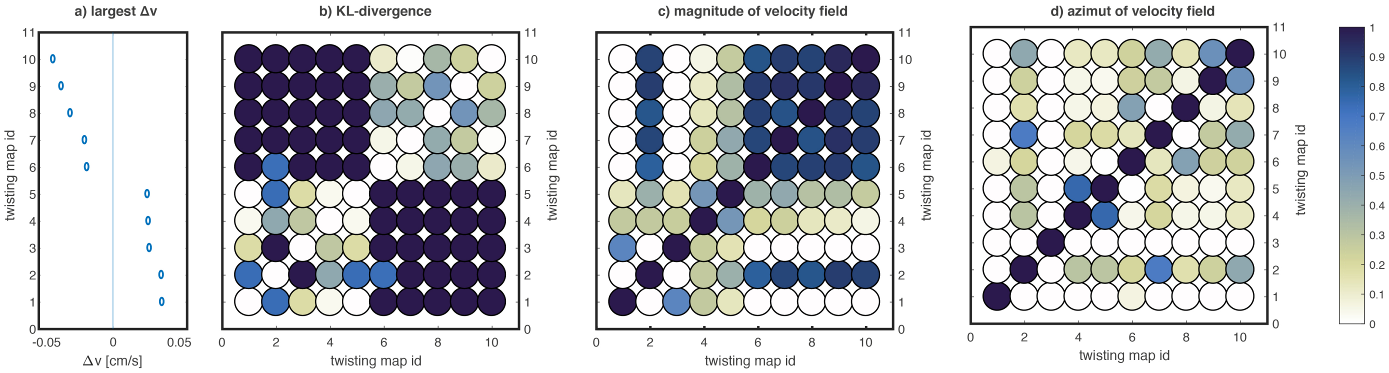

Supplementary Figure 5: Additional attempts to constrain the similarity between couples of twisting maps: **a** ten selected twisting maps showing the largest Δvs (likely those with the clearest signal). **b** we computed the Kullback-Leibler divergence (KL-div) for all combinations of couples of twisting maps. Couples of distributions have been created using the velocity field in the receiver region. Low values of KL-div indicate the selected couple of twisting map has high degree of similarity. Panel b shows twisting maps associated to positive Δvs result in low KL-div values when compared with twisting maps with positive Δv and high KL-div values when compared with twisting maps with negative Δv, and vice versa. In such representation, KL-div does a decent job and separates well 4 quadrants, however the spatial information (i.e., the precise pixel where velocity is extracted) is lost. To prevent this problem we also report two additional twisting similarity quantification strategies: **c** spatial correlation between maps of amplitude of velocity; **d** and spatial correlation between couples of maps of strike of the velocity. Dark blue markes in panel c and d indicate couples of twisting maps with high similarity (correlation). In both cases however we did not manage to separate well couples of accelerating couples of twisting maps versus decelerating ones, likely due to the fact that all analog ruptures are slightly different.

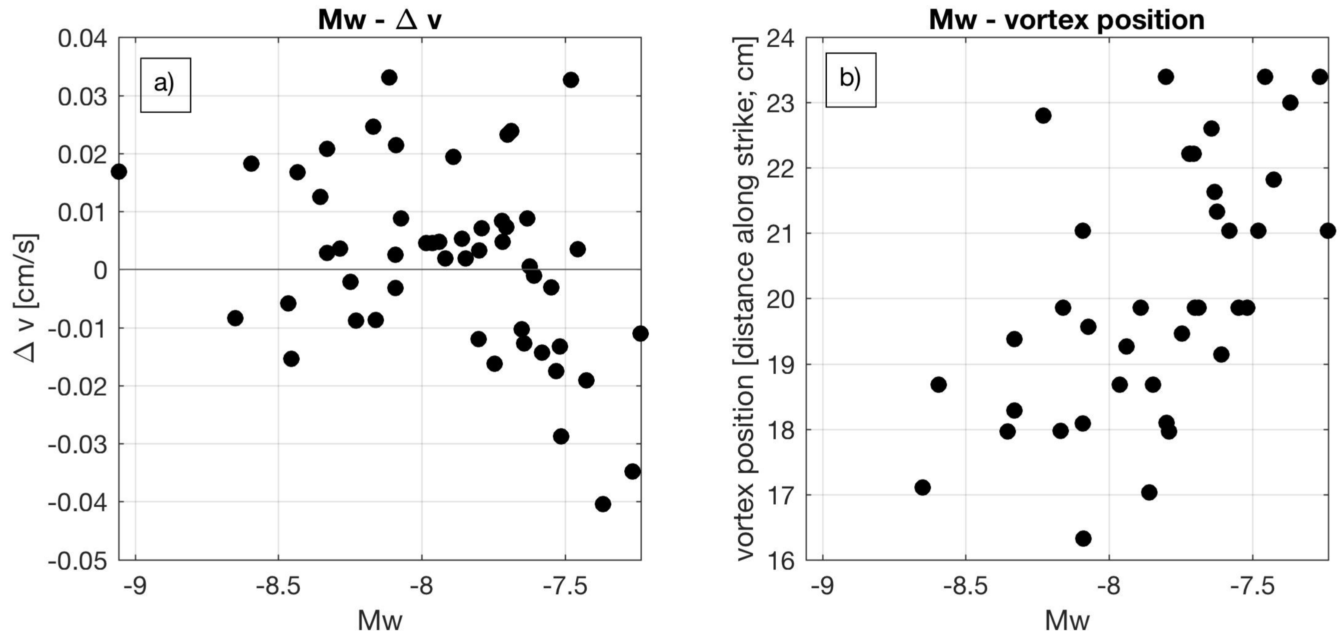

Supplementary Figure 6: Analog earthquakes magnitude Mw effect on receiver accelerations Δv and position of the twisting centers: we measured the area of the slipping region (A), the average displacement (d) and calculated the moment (Mo) of individual labquake as follow Mo=A*d*G; where for G we used 5000 Pa. Then we computed Mw as follow: Mw(j)=0.66*(log10(Mo(j)*1e7))-10.7; where j represents the event number and the multiplying factor 1e7 is used for N-m to dyne-cm conversion. **a** bivariate plot showing the relationship between Mw and Δv. **b** bivariate plot showing the relationship between Mw and the along strike position of twisting centers. This figure shows that Mw plays a role in controlling where the vortex center is located (the larger the events the further into the receiver asperity the centers) but it is not effective in controlling its spatial pattern in particular in the coastal receiver area (which on the contrary is controlled by the loading history).

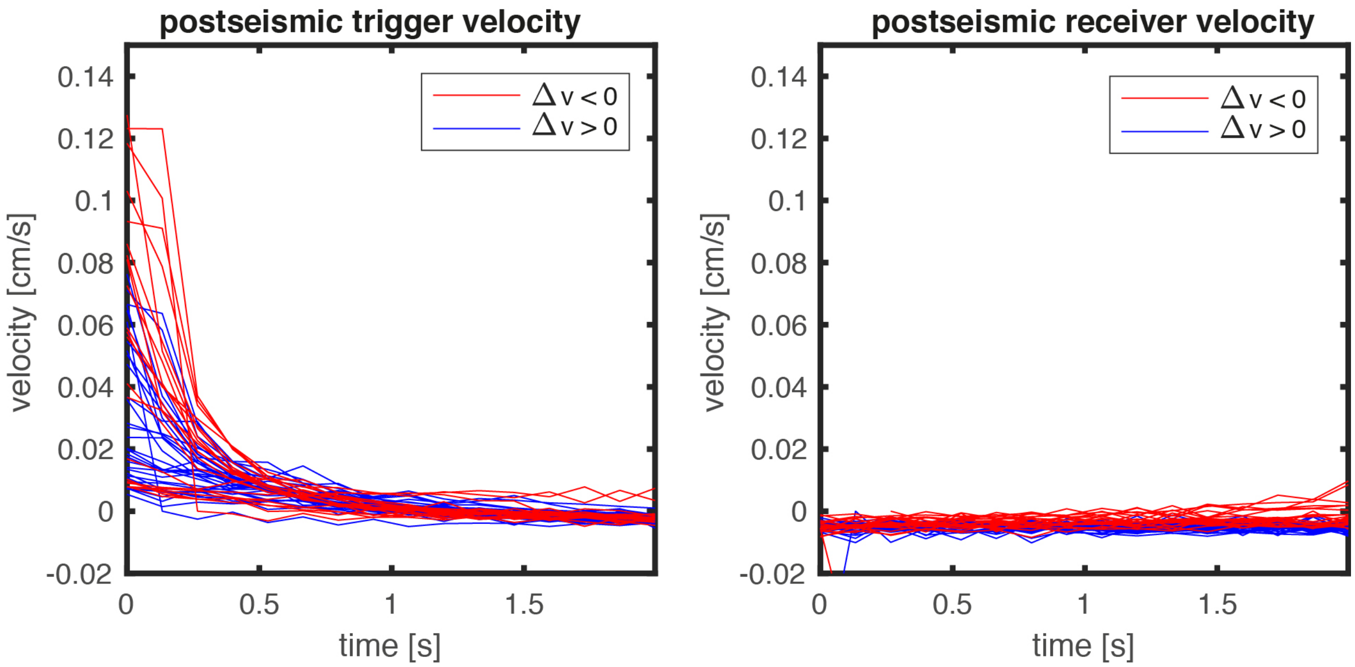

Supplementary Figure 7: Postseismic velocity from analogue subduction model. Figure showing postseismically decaying velocities in the trigger asperity and sustained velocities in the receiver asperity. In both panels, the horizontal axis represents time since the velocity peak in the trigger asperity.

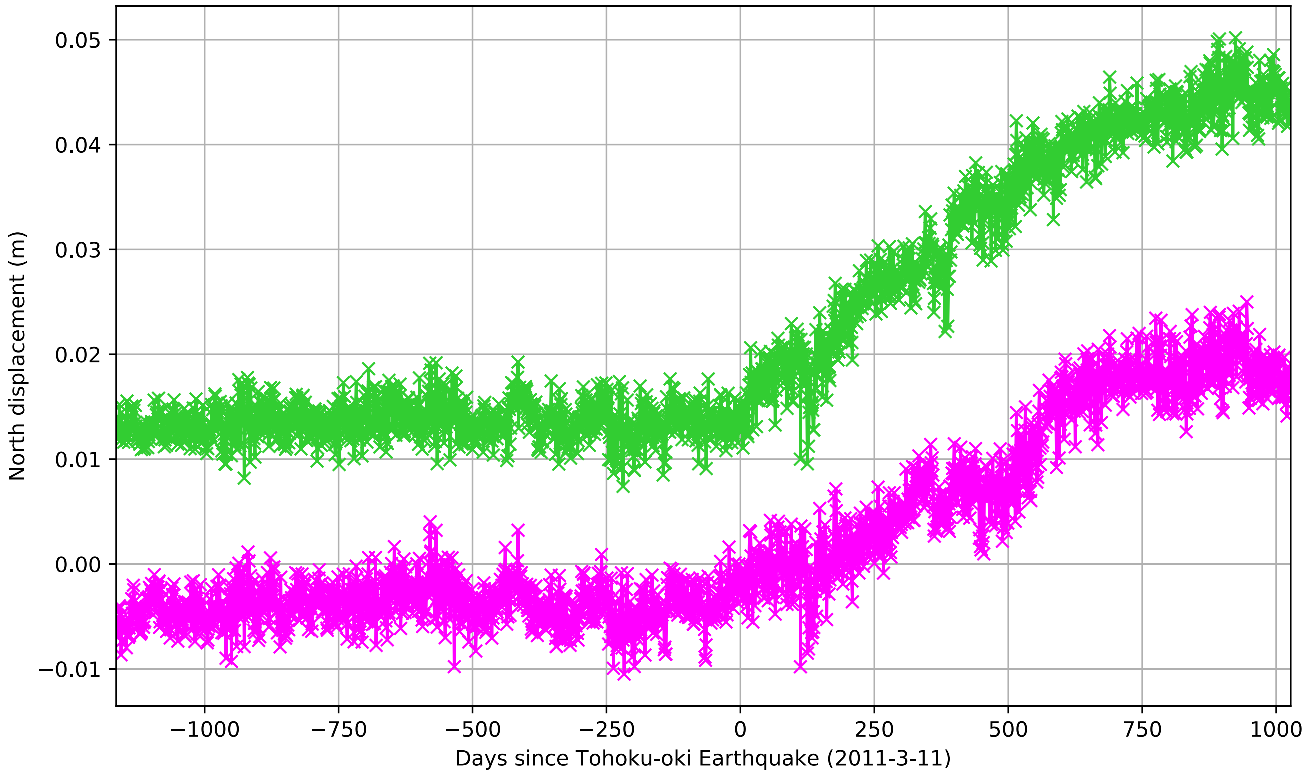

Supplementary Figure 8: Acceleration after Tohoku-oki earthquake. Green and fuchsia time series correspond to the stations with these colours on Supplementary Figure. 10b. Shown are the PPP GNSS displacement time series for the north component of each station, with steady-state seasonal and steps subtracted. Both time series have been detrended by the velocity calculated for the interseismic (time prior to the Tohoku-oki earthquake).

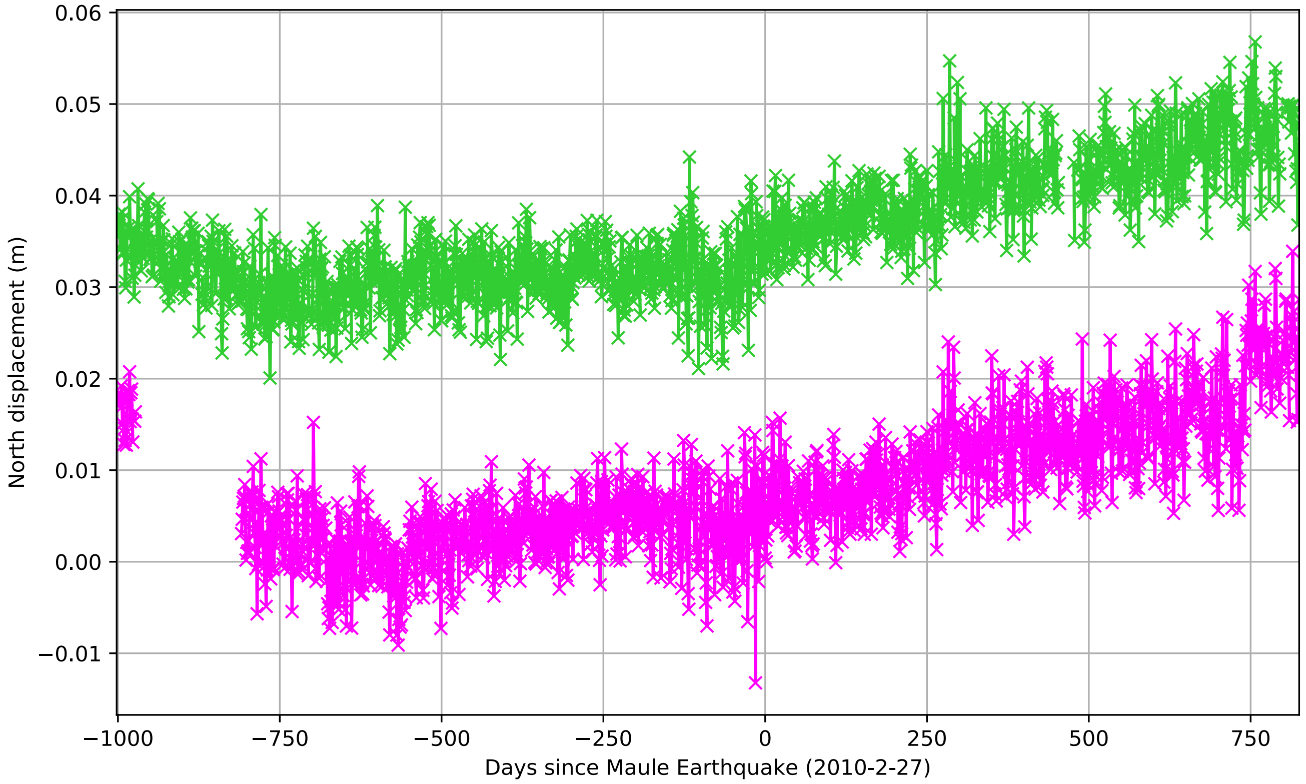

Supplementary Figure 9: Acceleration after Maule earthquake. Green and fuchsia time series correspond to the stations with these colours on Supplementary Figure 2b. Shown are the PPP GNSS displacement time series for the north component of each station, with steady-state seasonal and steps subtracted. Both time series have been detrended by the velocity calculated for the interseismic (time prior to the Maule earthquake).

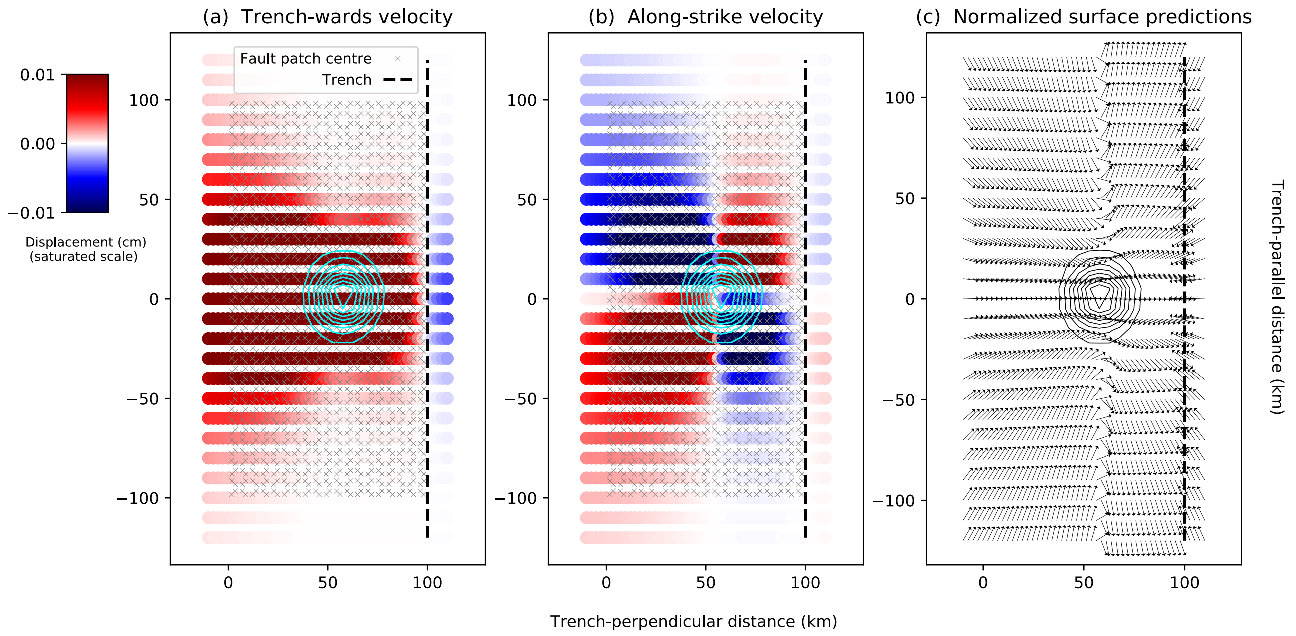

Supplementary Figure 10: Elastic dislocation model. All panels show the horizontal displacement field from an updip coseismic slip. Contours indicate the slip (from 0.2 to 1m in 0.1 m increments). On panels (a) and (b), the colourscale indicates the displacement in trench-wards and along-strike directions, respectively. The trench is indicated by the thick dashed line on the right of each plot, and the fault-patch centroids are given by the grey crosses in panels (a) and (b). **c** shows the unit vectors on the surface. From this figure, we see that the elastic dislocation model is unable to recreate the vortex-like twisting pattern that is observed in the laboratory and nature (see Figure 1 in main article file).

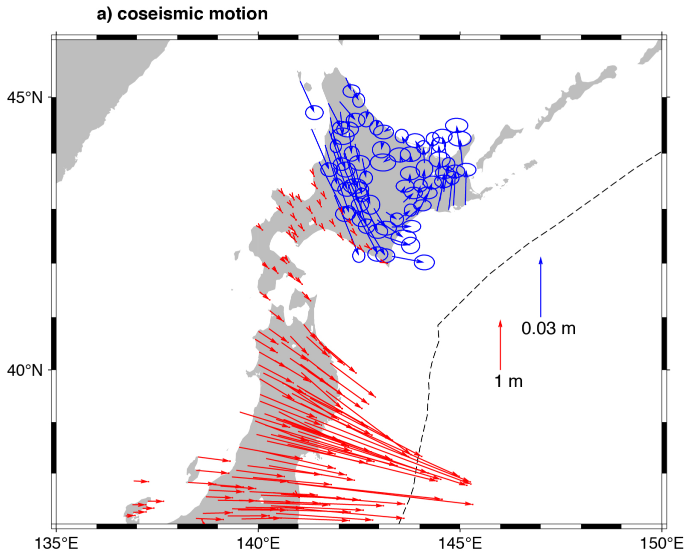

Supplementary Figure 11: Displacement of the Tohoku-oki earthquake. a, The coseismic motion of the Tohoku-oki earthquake. **b** The acceleration from interseismic to postseismic velocity. Due to much larger displacements in the vicinity of the Tohoku-oki rupture zone, we employ two colour scales. In both panels, we clearly see a vortex-like twisting pattern. Dashed line indicates the subduction trench. Ellipses represent 95% confidence.

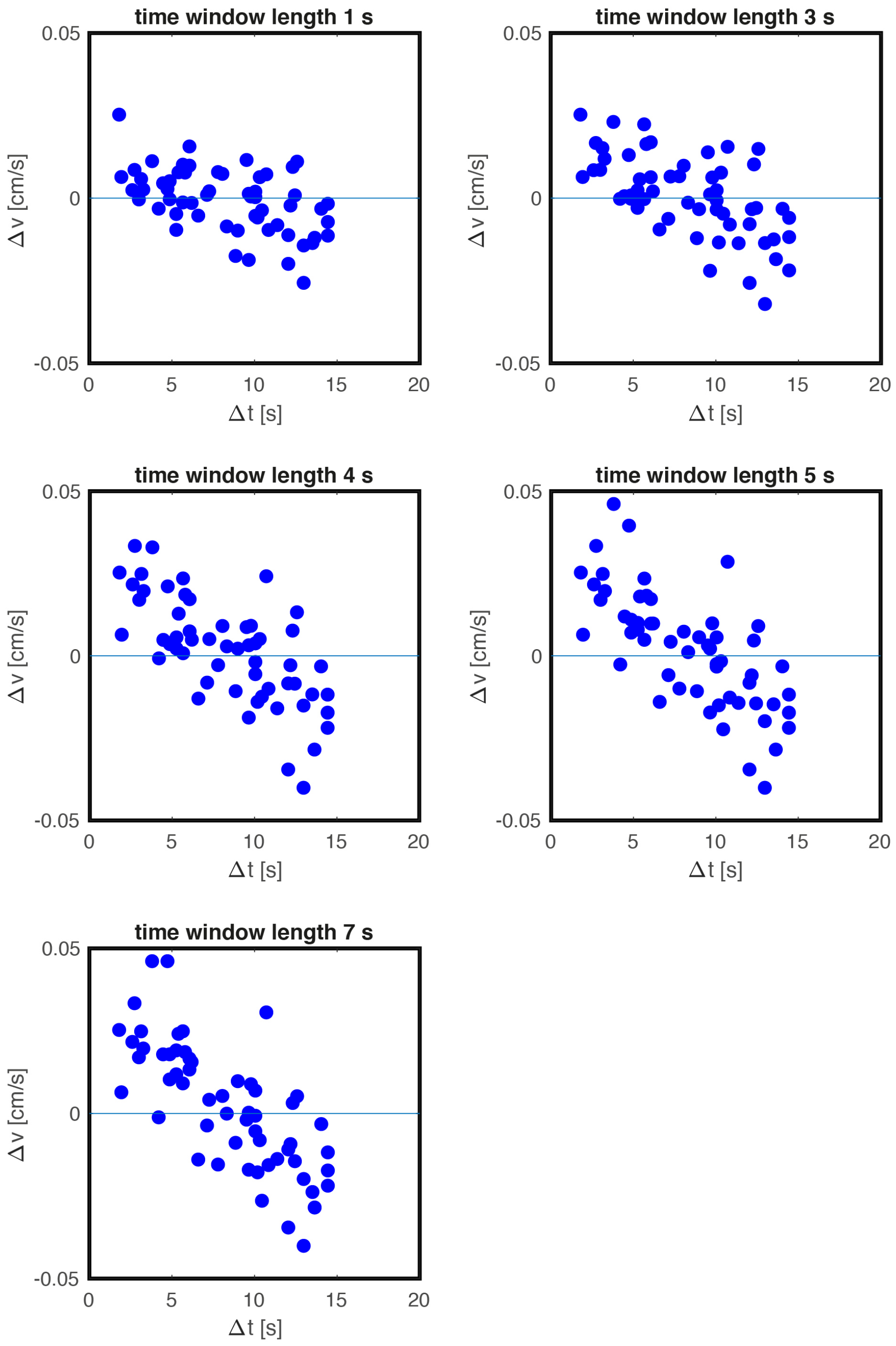

Supplementary Figure 12: Impact of analysis time window length on Δv - Δt proportionality. Each plot represents a time window of different length (reported in each title). Time windows larger than 7 s would lead to no data to plot as none of the interactions would satisfy the condition that the receiver patch does not slip during the analyzed time interval (i.e., linear fits computed for establishing interseismic velocity changes would be corrupted by coseismic displacement of the receiver).

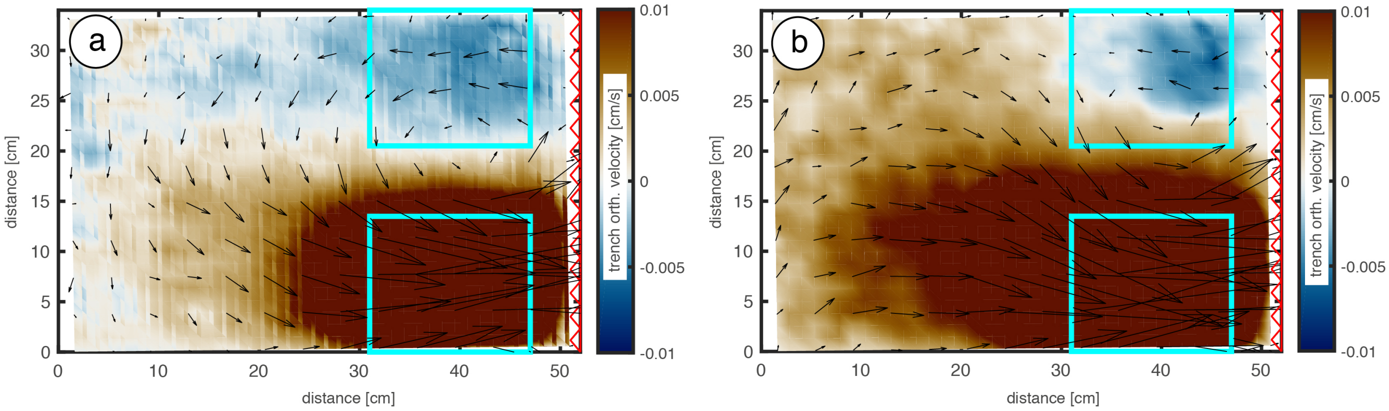

Supplementary Figure 13: Surface velocity during coseismic interaction: Trench orthogonal component of the velocity field is reported with the background shading for Δv>0 and Δv<0 interactions (panel a and b, respectively). Quiver showing velocities direction is also shown with density of virtual stations equal to 1/3 of the available dataset. Interactions are the same as those shown in figure 3 a and b in the main article file.
